# ADIPOSITY CHANGE IN ADULTS: THE IMPACT OF TRAIT NEUROTICISM

**DOI:** 10.1093/geroni/igac059.2375

**Published:** 2022-12-20

**Authors:** Jacob Alderson, Meredith Willard, Nicholas Turiano

**Affiliations:** West Virginia University, Morgantown, West Virginia, United States; West Virginia University, Morgantown, West Virginia, United States; West Virginia University, Morgantown, West Virginia, United States

## Abstract

Increased body weight is a risk factor for poor health and shortened life expectancy. Thus, it is imperative to understand how body weight changes across adulthood and to identify factors that predict weight gain so effective prevention strategies can be implemented. It is well-known that eating habits and physical activity are two of the most important factors (along with genetic factors) contributing to weight gain. However, we seek to determine if personality levels predict weight gain because individual differences in personality are thought to be the root-cause of many behaviors related to weight gain. We utilized longitudinal data on over 6,000 adults (aged 20-75 at baseline) from the Midlife Development in the U.S. Study (MIDUS). The Big 5 personality traits, body weight, waist circumference, and body mass index were measured three times from 1995-2015. We estimated a growth curve model to determine whether each adiposity measure changed over 20 years, controlling for age, gender, and education. There was a significant increase in all adiposity measures over time. The rate of adiposity change over time varied among persons (random: weight b = 0.543; waist b = 0.008; BMI b = 0.009; p values < .05). Higher levels of neuroticism predicted this variability (fixed: weight b = 0.211; waist b = 0.027; BMI b = 0.029; p values < .05) such that those scoring higher in trait neuroticism had a steeper increase in all three adiposity measures. These findings suggest that personality traits are important in the progression of weight-gain in adults.

